# Supplementary material for: Differentiation of Murine C2C12 Myoblasts Strongly Reduces the Effects of Myostatin on Intracellular Signaling
Source: Biomolecules. 2020 Apr 30;10(5):695. doi: 10.3390/biom10050695 (PMC7277184; doi:10.3390/biom10050695)
Supplement: Supplementary file 1 [file biomolecules-10-00695-s001.pdf]

Supplementary Material:

Differentiation of murine C2C12 myoblasts strongly decreases their responsiveness to myostatin

Juulia H. Lautaoja, Satu Pekkala, Arja Pasternack, Mika Laitinen, Olli Ritvos and Juha J. Hulmi

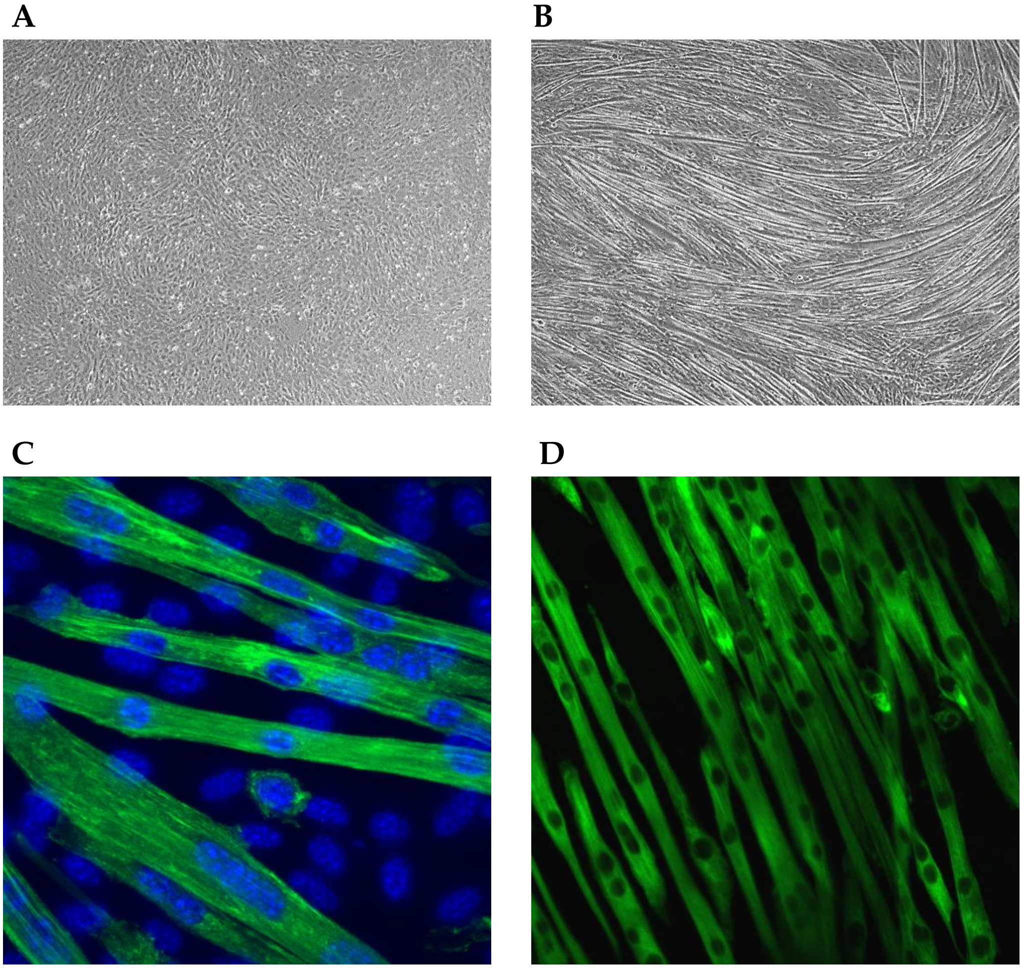

FIGURE S1. Representative images of the C2C12 cells. A) 5X bright field image of C2C12 myoblasts and B) C2C12 myotubes. C) 63X and D) 10X immunofluorecent images of the C2C12 myotubes. C2C12 myoblasts were grown and differentiated on glass coverslips as described in material and methods section. At day 5 post differentiation, the cells were washed with PBS, fixed in -20°C acetone for 10 min and stored in PBS at +4°C for a maximum of one week before staining. The cells were permeabilized with 0.5% Triton-X-100 for 10 min at room temperature (RT), blocked with a mixture of 10% normal goat serum, 1% bovine serum albumin and 0.1% Triton-X-100 in PBS for 1h RT, washed with PBS and incubated for 1h at RT with primary antibody in blocking solution. After PBS washes, Alexa fluorochrome conjugated secondary antibody (Goat anti-mouse Alexa Fluor 488) was incubated for 1h at RT followed by PBS washes and DAPI staining 10 min RT (not in D). Finally, samples were imaged using a Zeiss LSM 700 confocal microscope. The antibodies used are presented in the Supplementary Material (Table S1).

TABLE S1. Antibodies used in western blot (WB) and immunocytochemistry (ICC).

Antibody	Manufacturer	Catalogue number	Dilution
Phosphorylated Smad 3$^{Ser423/Ser425}$	Abcam	ab52903	WB 1:1000
Total Smad 3	Abcam	ab40854	WB 1:1000
Phosphorylated p70/S6K1/2^{Thr389}	Cell Signaling Technology	#9234	WB 1:1500
Total p70/S6K1/2	Cell Signaling Technology	#2708	WB 1:1000
Phosphorylated p38$^{Thr180/Tyr182}$	Cell Signaling Technology	#4511	WB 1:1000
Total p38	Cell Signaling Technology	#9212	WB 1:1000
Phosphorylated p44/42 MAPK (ERK1/2)$^{Thr202/Tyr204}$	Cell Signaling Technology	#9101	WB 1:1000
Total p44/42 MAPK (ERK1/2)	Cell Signaling Technology	#9102	WB 1:1000
Phosphorylated Akt^{Ser473}	Cell Signaling Technology	#9271	WB 1:1000
Total Akt	Cell Signaling Technology	#4691	WB 1:1000
Phosphorylated C/EBPβ^{Thr235}	Cell Signaling Technology	#3084	WB 1:1000
Total C/EBPβ	Cell Signaling Technology	#3082	WB 1:1000
Phosphorylated S6$^{Ser240/Ser244}$	Cell Signaling Technology	#2215	WB 1:2000
Total S6	Cell Signaling Technology	#2217	WB 1:2000
Phosphorylated SAPK/JNK$^{Thr183/Tyr185}$	Cell Signaling Technology	#4668	WB 1:1000
SAPK/JNK	Cell Signaling Technology	#9252	WB 1:1000
Phosphorylated Stat 3^{Tyr705}	Cell Signaling Technology	#9145	WB 1:1000
Total Stat 3	Cell Signaling Technology	#9139	WB 1:1000
MF-20-c*	Developmental Studies Hybridoma Bank	AB 2147781	ICC 1:100
Follistatin	LSBio	LS-B14665	WB 1:1000
Puromycin	Merck	MABE343	WB 1:4000
Ubiquitin	Santa Cruz Biotechnology	sc-8017	WB 1:700

* MF 20 was deposited to the DSHB by Fischman, D.A. (DSHB Hybridoma Product MF 20)

For western blotting, secondary antibodies used were horseradish peroxidase-conjugated secondary IgG antibodies (Jackson ImmunoResearch Laboratories, PA, USA) (diluted anti-rabbit 1:10 000, anti-mouse 1:30 000).

TABLE S2. Primers used in real-time qPCR analyses.

Transcript	Forward sequence, 5´- 3´	Reverse sequence 5´- 3´	Bio-Rad Assay ID
ACVR2B, human			qHsaCID0016227
Acvr2b, mouse			qMmuCID0015599
FOLLISTATIN, human			qHsaCID0014487
Follistatin, mouse			qMmuCID0022360
GDF8 (myostatin), human	TGGTCATGATCTTGCTGTAACC	CTTGACCTCTAAAAACGGATTCA	
Gdf8 (myostatin), mouse	AAGATGGGCTGAATCCCTTT	GCAGTCAAGCCCCAAAGTCTC	
GDF11, human	ACCACCGAGACCGTCATTAG	AGGGCTGCCATCTGTCTG	
Gdf11, mouse	CGTCACATCCGTATCCGTTC	AAAGGATGCAGCCCCTCAG	
INHIBINβA, (activin A), human	GCTCAGACAGCTCTTACCACA	AGCAAATTCTCTTTCTGGTCCC	
InhibinβA (activin A), mouse	GAACGGGTATGTGGAGATAG	TGAAATAGACGGATGGTGAC	

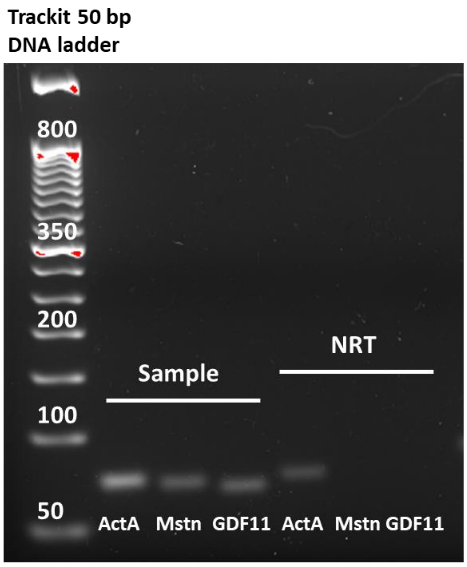

FIGURE S2. Amplicon length of the RT-qPCR products in human CHQ cells. Amplicon length of Activin A (ActA, 72 bp), Myostatin (Mstn, 70 bp) and GDF11 (70 bp) were tested on agarose gel. The product size of all the primers were as expected. Primer sequences are presented in table S1. NRT = no reverse transcript control.

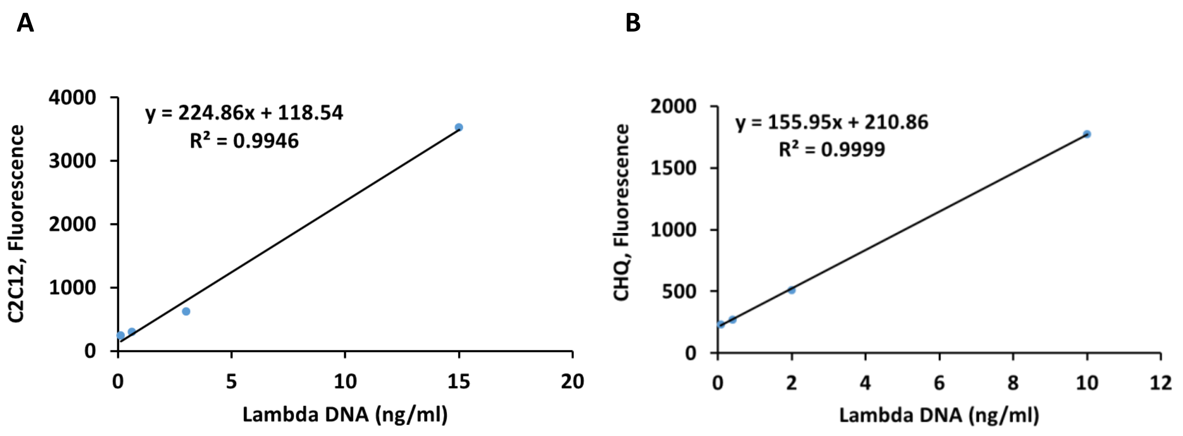

FIGURE S3. PicoGreen normalization of the RT-qPCR results. (A) standard-curve and equation for the C2C12 cells. (B) standard-curve and equation for the CHQ cells.

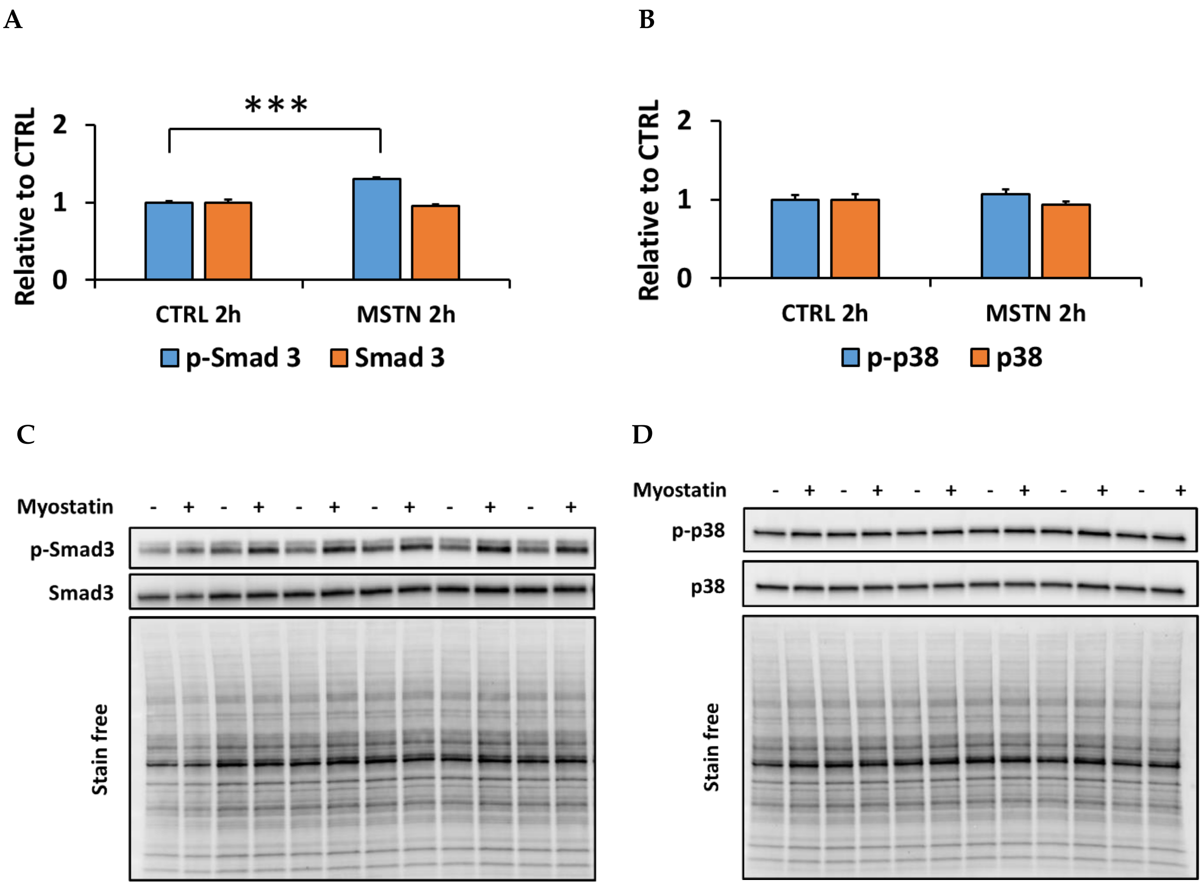

FIGURE S4. Repeated 2h C2C12 myotube experiment with alternative differentiation protocol (2% horse serum, HS). Despite differentiation protocol, similar results were obtained as the results of 5% fetal bovine serum (FBS) differentiation demonstrating that the response of the myotubes to 100 ng/ml myostatin is not affected by used differentiation protocol. (A) Phosphorylated Smad3$^{Ser423/425}$ and total Smad3. (B) Phosphorylated p38$^{Thr180/Tyr182}$ and total p38. (C-D) Representative blots. In the figures, the values are presented as normalized to CTRL = 1. *N* = 6 per group, *** = *P* < 0.001.

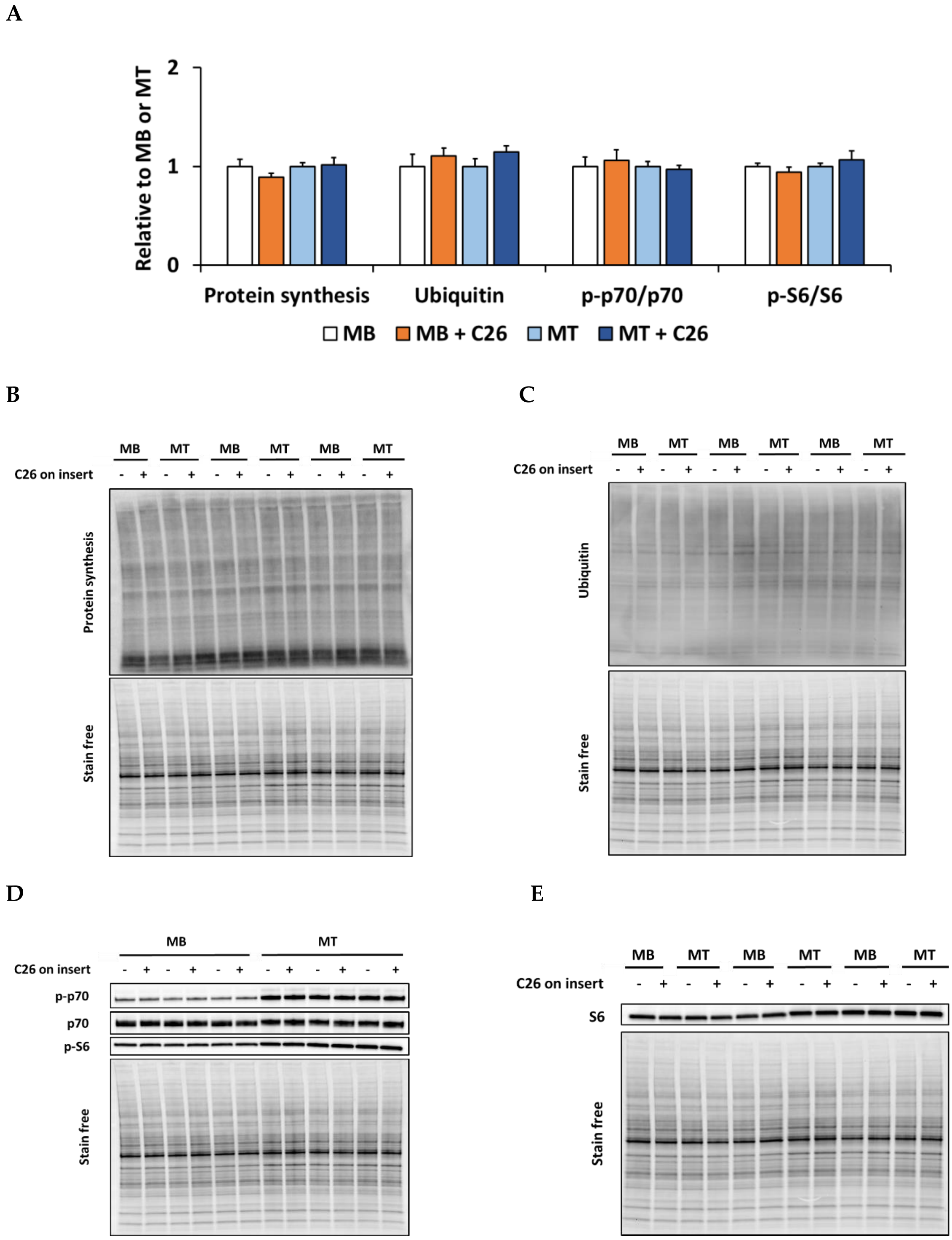

FIGURE S5. 24-hour co-culture of C2C12 myoblasts and myotubes with C26 cancer cells does not affect (A) protein synthesis measured by SUnSET method, proteolysis (ubiquitin) or mTOR signalling analysed as phosphorylated S6K1/2 ($p70^{Thr389}$) per total S6K1/2 (p70) and phosphorylated ribosomal protein S6 ($S6^{Ser240/244}$) per total S6. In histograms, myoblasts (MB) or myotubes (MT) with empty insert are set as one and compared with MB or MT with C26 cells on the insert (MB+C26 and MT+C26, respectively). (B-E) Representative blots. *N* = 5-8 per group.

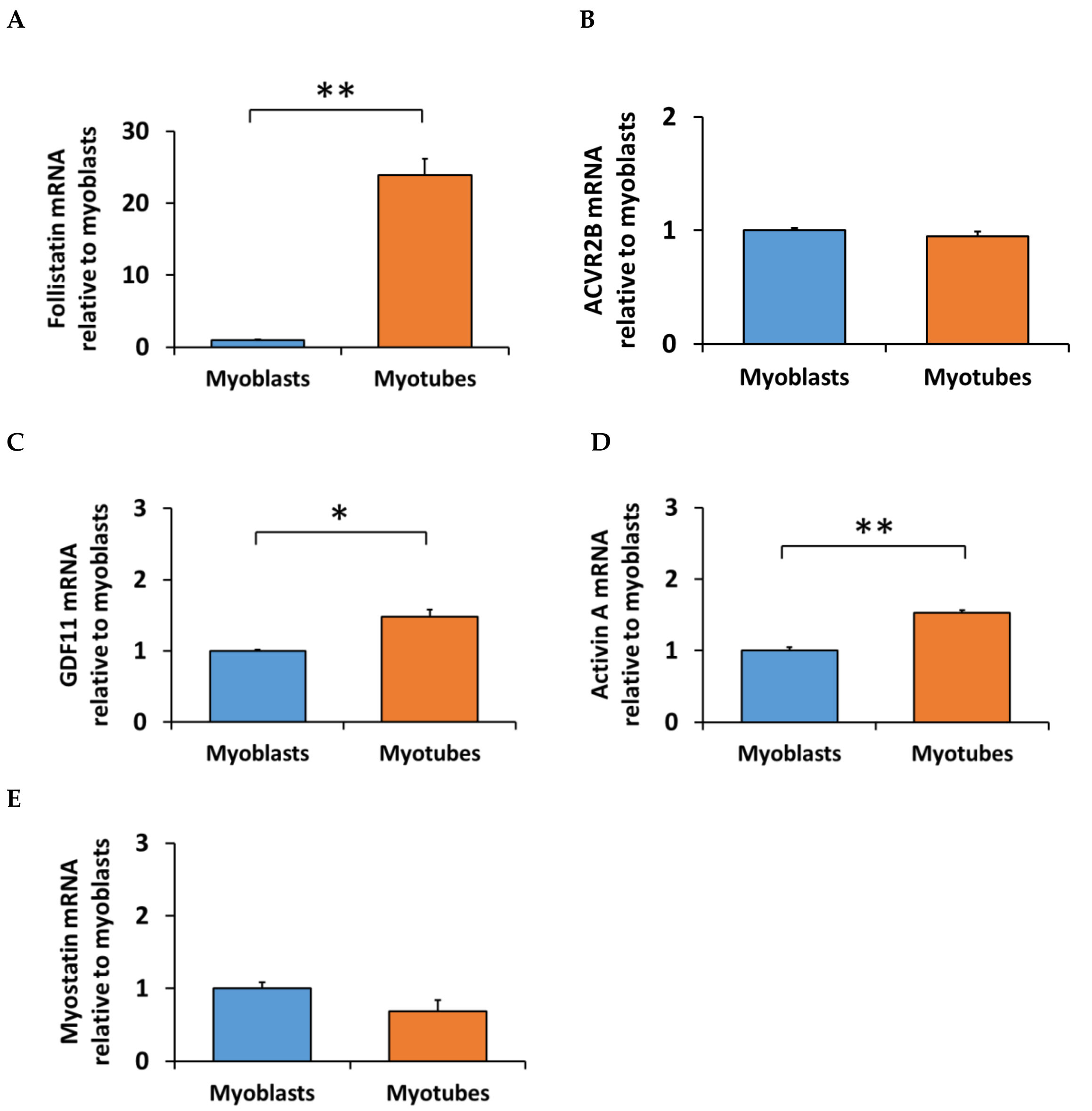

FIGURE S6. Repeated 2h C2C12 myotube experiment with alternative differentiation protocol (2% horse serum, HS). Despite the differentiation protocol used, similar mRNA results were obtained as the results of 5% fetal bovine serum (FBS) differentiation. mRNA level (A) follistatin, (B) ACVR2B, (C) GDF11, (D) activin A and (E) myostatin in C2C12 myoblasts and myotubes differentiated with 2% HS. In the figures, the values are presented as normalized to myoblasts = 1. *N* = 2-3 per group. * and ** = $P < 0.05$ and $P < 0.01$, respectively.

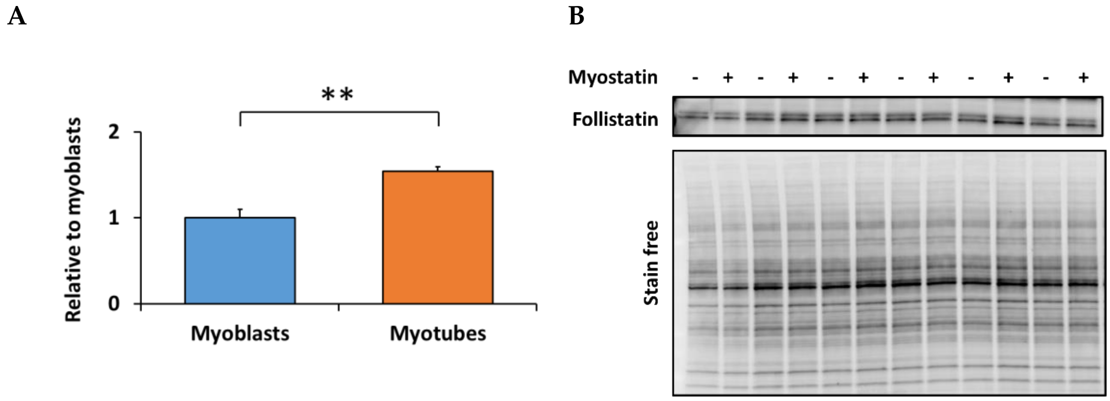

FIGURE S7. Protein content of (A) follistatin increased also in the myotubes differentiated with 2% HS. In the figure, the values are presented as normalized to myoblasts = 1. (B) Representative blot. $N = 12$ per group as treated and non-treated groups were pooled due to lack of myostatin effect. ** = $P < 0.01$.
